# Disproportionate positive feedback facilitates sense of agency and performance for a reaching movement task with a virtual hand

**DOI:** 10.1371/journal.pone.0233175

**Published:** 2020-05-20

**Authors:** Raviraj Nataraj, David Hollinger, Mingxiao Liu, Aniket Shah

**Affiliations:** 1 Movement Control Rehabilitation (MOCORE) Laboratory, Stevens Institute of Technology, Hoboken, NJ, United States of America; 2 Department of Biomedical Engineering, Stevens Institute of Technology, Hoboken, NJ, United States of America; University of Milan, ITALY

## Abstract

This study investigated the generalized effects of positive feedback (PF) versus negative feedback (NF) during training on performance and sense of agency for a reach-to-touch task with a virtual hand. Virtual reality (VR) is increasingly employed for rehabilitation after neuromuscular traumas such as stroke and spinal cord injury. However, VR methods still need to be optimized for greater effectiveness and engagement to increase rates of clinical retention. In this study, we observed that training with disproportionate PF subsequently produced greater reaching performance (minimizing path length) and greater agency (perception of control) than with disproportionate NF. During PF training, there was also progressive increase in agency, but conversely a decrease in performance. Thus, the increase in performance after training may not be due to positively bolstered learning, but rather priming higher confidence reflected in greater agency. Agency was positively measured as compression in perceived time-intervals between the action of touch to a sound consequence, as standard with intentional binding paradigms. Positive feedback desirably increased agency (~180 msec) and reduced path length (1.8 cm) compared to negative feedback, which itself showed insignificant, or neutral, effects. Future investigations into optimizing virtual reality paradigms for neuromotor rehabilitation should consider agency as a driving factor for performance. These studies may serve to optimize how feedback is better presented with performance results for complex motor learning. Investigators should also ponder how personal characteristics, both cognitive and physical, may further affect sensitivity to feedback and the rate of neuromotor rehabilitation.

## 1 Introduction

Feelings of reward orchestrate a chorus of neural processes useful for directing performance of a desired outcome. Prior research substantiates how reward influences performance expectancy and autonomy (self-efficacy) during goal-oriented tasks [1]. The underlying neural pathways activated by reward can also be leveraged for sensorimotor learning of movement [2]. Reward can activate dopaminergic projections from the midbrain to the primary motor cortex to encode motor skill [3, 4]. Reward-based movement training is posited to facilitate sensorimotor learning during adaptation, activity-dependent plasticity, and skill learning [5, 6]. Predictive models of motor control purport that reward promotes enhanced expectancies for optimizing skill [1]. Advanced rehabilitation paradigms, employing robotics or virtual reality (VR), have incorporated reward feedback to accelerate neurorehabilitation [7–9]. Neuromuscular traumas requiring rehabilitation to restore abilities to perform activities of daily living (ADLs) include spinal cord injury (SCI, 11,000 persons per year in U.S. [10]), traumatic brain injury (TBI, 1.5 million per year [11]), amputation (185,000 per year [12]), and stroke (800,000 per year [13]).

Physical therapy following neuromuscular trauma often involves repetitive task activity to restore and functionally readapt neural pathways [9]. Physical therapy helps patients to re-train or re-learn basic motor skills such as reaching and grasping, and then achieve greater independence. To date, these techniques mainly focus on building physical strength and skill rather than psychological and cognitive aspects of motor learning. Thus, patients may feel less integrated with rehabilitation procedures, leaving them frustrated and more likely to abandon such tools [14] or methods [15] altogether. Virtual reality interfaces can motivate greater commitment and better motor outcomes through cognitive engagement [16]. Virtual reality facilitates engagement with colorful visual displays and rhythmic audio feedback [17, 18], personalization [19], or incentivizing progress with reward, as with gamification [20]. While reward is implicated with sensorimotor learning and motivation, its effects on perception of movement control is unclear. Sense of agency is feeling of control over actions, such as movements, and related consequences [21]. Implicit (subconscious) agency with movement initiation has been positively associated with states of higher arousal [22]. Attention is a critical factor for cognitive engagement and adherence to rehabilitation practices [23]. Although agency merges cognitive attention with motor control, little research has been conducted to directly relate feedback intended to motivate with agency and performance. Due to the customization capabilities within computerized environments, rehabilitation using virtual reality can be an especially powerful tool to investigate and implement optimal forms of feedback. To expand rehabilitation potential, optimal VR feedback would motivate not only greater engagement and commitment to a protocol, but also facilitate greater agency to accelerate actual movement performance.

Sense of agency has been studied in experiments that match actions to expected consequences [24, 25], observe modulation of agency with external cues [26], and examine the existence of agency with human-computer-interactions [27]. Agency is implicated with rehabilitation through perception of neuromuscular action and functional consequences [28]. Agency contributes to the execution of functional movements such as reaching and grasping during ADLs that engage the environment [29]. Previous studies have shown that neurological disorders can impair agency [30, 31]. Neuromuscular deficits naturally compromise agency as affected persons can attempt functional movements, but without desired consequences. In persons that utilize powered assistive devices, such as exoskeletons [32, 33] or sensorimotor prostheses [34–36], agency may be compromised due to distortions in embodiment [37, 38]. However, it remains unclear how modulating agency could affect the functional movement performance. If clearer connections between agency and functional performance are established, further studies on enhancers of movement agency could markedly improve rehabilitation protocols on cognitive levels.

The phenomenon of intentional binding is an implicit measure for agency. It indicates how coupled in time one perceives an intended action to an expected sensory consequence during voluntary control [29]. Time-interval estimation between action and consequence is now a standard basis from which to implicitly infer agency from intentional binding. Participants would judge the time duration between an action (e.g., key press) and sensory consequence (e.g., sound tone). In the seminal work [29], a perceptual *compression* of time was observed when the action was voluntary (high agency) versus an involuntary (low agency) twitch induced by transcranial magnetic stimulation. Intentional binding has been used to show the influence of sensorimotor processes on agency through internal prediction and external action outcomes [39, 40]. Time-interval estimation paradigms [28] are well suited to study cognitive-performance effects of reward and sensory cues for VR-based motor rehabilitation [41, 42].

Investigating other cognitive factors such as psychology and personality may also provide insight into more effective rehabilitation methods. Patients with neuromuscular deficits often suffer from psychological feelings of depression, anxiety, and hopelessness [43]. Personality traits have been investigated in their influence on individual health and rehabilitation outcomes [44]. *Internals* are persons not as affected by extrinsic factors, primarily believe major health outcomes are dictated by personal choices. They tend to progress better in rehabilitation compared to *externals*, who feel less independence and power in controlling personal health outcomes. It would follow that internals are not as affected by extrinsic feedback, like reward. Externals are more likely influenced by, if not seeking of, external sources of behavioral reinforcement.

External reinforcement can have operant effects of behavior as described in Skinner’s model examining the probabilistic strengthening by reinforcement [45]. Alternatively, the mechanistic effects of simple positive feedback, serving as an inducement, for better movement and sense of movement agency remains unclear. Furthermore, how positive feedback is provided to necessarily serve as reward during motor behavior must also be carefully considered. The effects of positive, or negative, feedback on movement may depend on the mode of delivery of feedback, the experimental context, and the specific characteristics of the person receiving feedback. Feedback from an external source can improve future and immediate motor performance [18, 46]. Depending on personality type and tendencies, optimizing behavioral effects, as with movement rehabilitation, may require careful consideration of how performance feedback is best provided. Considerations include true value performance feedback for learning, reward for motivation, and optimizing feedback based on personality type. Thus, understanding the overarching effects of reward on cognition of movement may inform more effective design approaches for neuromuscular rehabilitation.

In this study, we investigated the relationship between positive feedback, sense of agency, and performance during a virtual upper limb reaching task. We hypothesized that disproportionate positive feedback (PF) produces improvement in functional performance *and* increase in agency compared to disproportionate negative feedback (NF). Our experimental construct provided feedback that was not strictly performance-based, but largely predetermined for feelings of affirmation or negation during task engagement. In this way, we better isolated feelings of motivation from learning effects precipitated by positive reinforcement of desired behavior [45]. Performance-based reward and punishment is known to enhance motor rehabilitation [47]. However, we chose to investigate and compare disproportionate PF versus NF, both of which can induce motivation for better performance [48, 49]. Reward serving as cognitive inducement for feelings of satisfaction is not the classical Skinnerian mode for positive reinforcement. Our objective was to examine whether simple PF or NF, both presumed to potentially motivate, can still precipitate greater motor performance in addition to greater agency. We further hypothesized that participants screened (by survey) for greater externality would be more affected by feedback.

Participants performed a reach-to-touch task in virtual reality with the primary goal of minimizing the reach path length to the target. Additional sub-goals instructed to the participants included matching the time of virtual touch contact with an observable speed pacer. Agency was measured from time-interval estimation between the action of touch contact and a proceeding sound beep. Simple positive (‘GOOD’) and negative (‘BAD’) message feedback for each trial was utilized to serve as presumptive positive and negative feedback. Unlike studies utilizing true performance feedback to enhance motor learning [50], the objective in this study was to demonstrate the effect of simple positive or negative tone with feedback on agency and performance of reaching. As such, regardless of actual performance, subjects were predetermined to receive a preponderance of positive or negative feedback depending on assignment to the reward or punishment cohort. We chose not to have a neutral feedback cohort as control since our task was simple to mitigate potential for learning and to specifically dissociate effects of feedback with desired or undesired performance [47].

## 2 Methods

**Fig 1:**
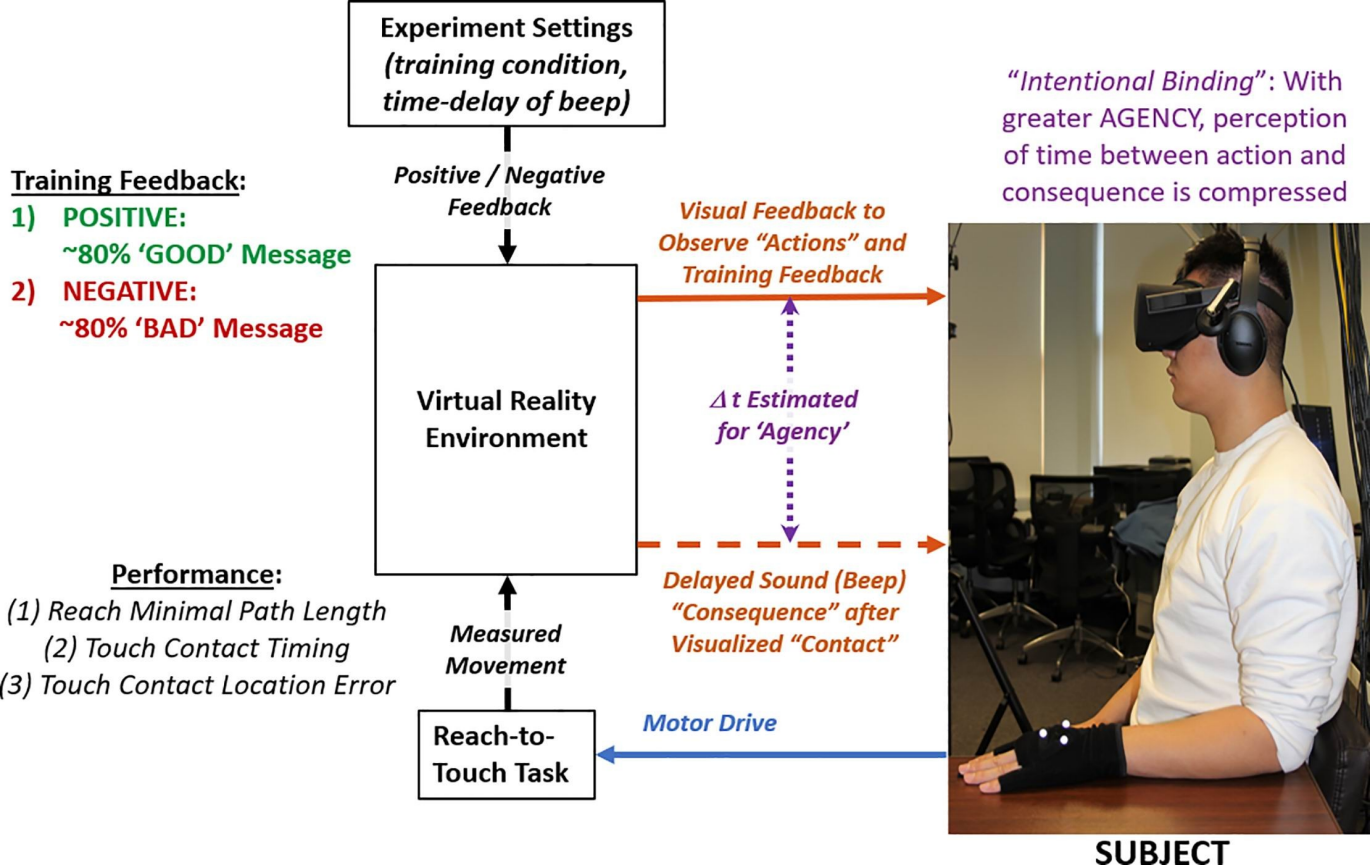
Flow diagram of experiment of participant performing reach-to-touch task with virtual hand under training feedback that is prescribed as either ‘positive’ or ‘negative’. Performance and agency are assessed with each reach-to-touch trial.

**Fig 1** shows an overview of the experimental protocol. Subjects performed a reach-to-touch task with a virtual hand while agency was assessed with either positive or negative feedback training. Disproportionate positive feedback training involved receiving a preponderance of the ‘GOOD’ message after each touch contact onto a target. Correspondingly, disproportionate negative feedback training involved receiving a preponderance of the ‘BAD’ message after each contact. For performance, subjects attempted to minimize path length of their reach, time their reach-to-touch, and accurately touch the center of each designated target. After the action of touch contact, the positive/negative message was immediately displayed and a sound (beep) was delay-executed as consequence at a variable time-interval. The subject estimated time-intervals as measurement for agency via intentional binding.

### Subjects

Twenty-four able-bodied volunteers were initially recruited to participate in this study. All subjects were right-handed to avoid consideration of hand dominance. There was no indication from preliminary analyses [51] that gender or age was a factor in this study. All subjects had normal or corrected-to-normal vision and did not report a history of diseases, or injuries affecting cognition or upper-extremity function. All subjects signed an informed consent approved by the Stevens Institute of Technology’s Institutional Review Board (protocol no. 2018–024), which also specifically approved this research study. Each session lasted less than 2 hours. Ultimately, 22 volunteers (Age = 21.0 ± 2.6, 19 Males, 3 Females) participated in the virtual reality protocol (see below for *Virtual Reach-to-Touch Task*) as two prospective volunteers were excluded due to inadequate ability to estimate time-intervals (see below for PRELIMINARY SCREENING).

### Equipment

**Fig 2:**
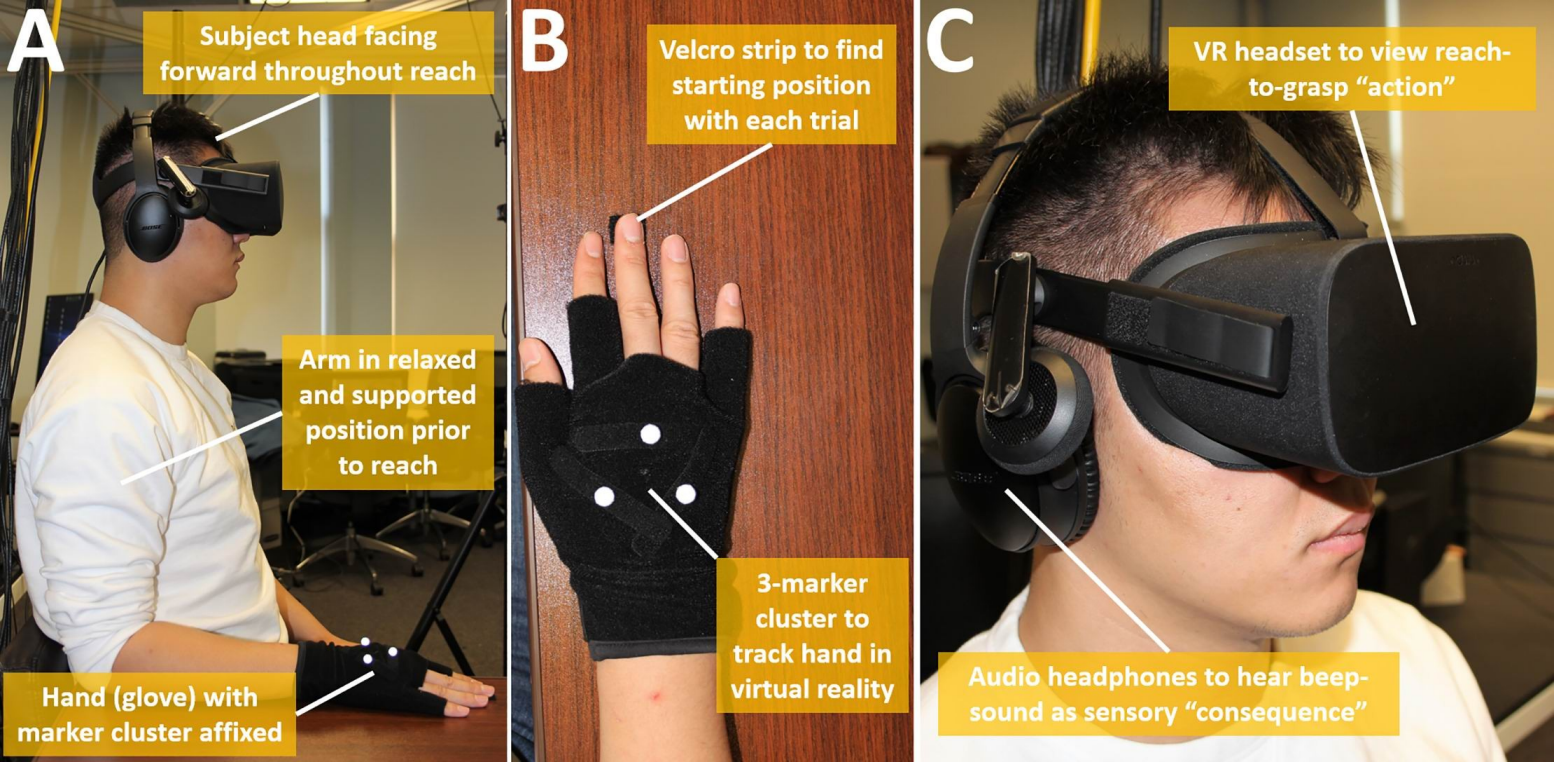
Experimental set-up elements–**A)** Subject body and arm position at start of each trial, **B)** Motion capture marker cluster affixed by Velcro on back of hand, **C)** Subject head with Oculus virtual reality headset and noise-cancelling headphones.

A motion capture system recorded 3-D hand movements of subjects to drive concurrent motion of a virtual prosthetic hand (MPL, *Modular Prosthetic Limb*, [52]). The hand was displayed in a 3-D virtual reality environment (MuJoCo, *Multi-Joint Dynamics with Contact*, Roboti LLC, Seattle, Washington, USA) [53]. Nine infra-red cameras (*Prime 17W* by Optitrack®, NaturalPoint Inc., Corvalis, OR, USA) were used to stream in real-time (120 Hz in *Motive* by Optitrack®) the 3-D position and orientation of a three-marker cluster on the back of the subject’s hand. Each retroreflective marker (9 mm diameter) was Velcro-affixed in a triangular arrangement (**Fig 2B**). Translation of real-time data to trigger virtual reality events was done using API code run in MATLAB® (Mathworks Inc., Natick, MA, USA). Participants wore an Oculus® *Rift* headset (Facebook Technologies, LLC) to view the virtual environment. Sound beeps for agency assessment were provided through a noise canceling headphone (Bose® *QuietComfort 35*, Framingham, Massachusetts).

### Protocol

#### Subject preparation

Upon arrival, subjects were asked to remove all personal jewelry and other worn reflective objects before being seated within the capture volume. The chair was height-adjusted to allow the subject to sit upright and position the elbow at 90 degrees flexion while the arm was weight supported by an adjacent table prior to each trial (**Fig 2A**). Each subject then donned the glove with marker-cluster affixed on back. The research assistant then carefully placed the VR headset, followed by audio headphone, onto the subject (**Fig 2C**). Each subject was instructed on how to find the starting position for their arm and hand on the table at the start of each trial. Instructions included: (1) rest the elbow, forearm, and hand on the adjacent table surface, and, (2) find and place the middle finger on a small patch of Velcro (**Fig 2B**).

#### Virtual reach-to-touch task

**Fig 3:**
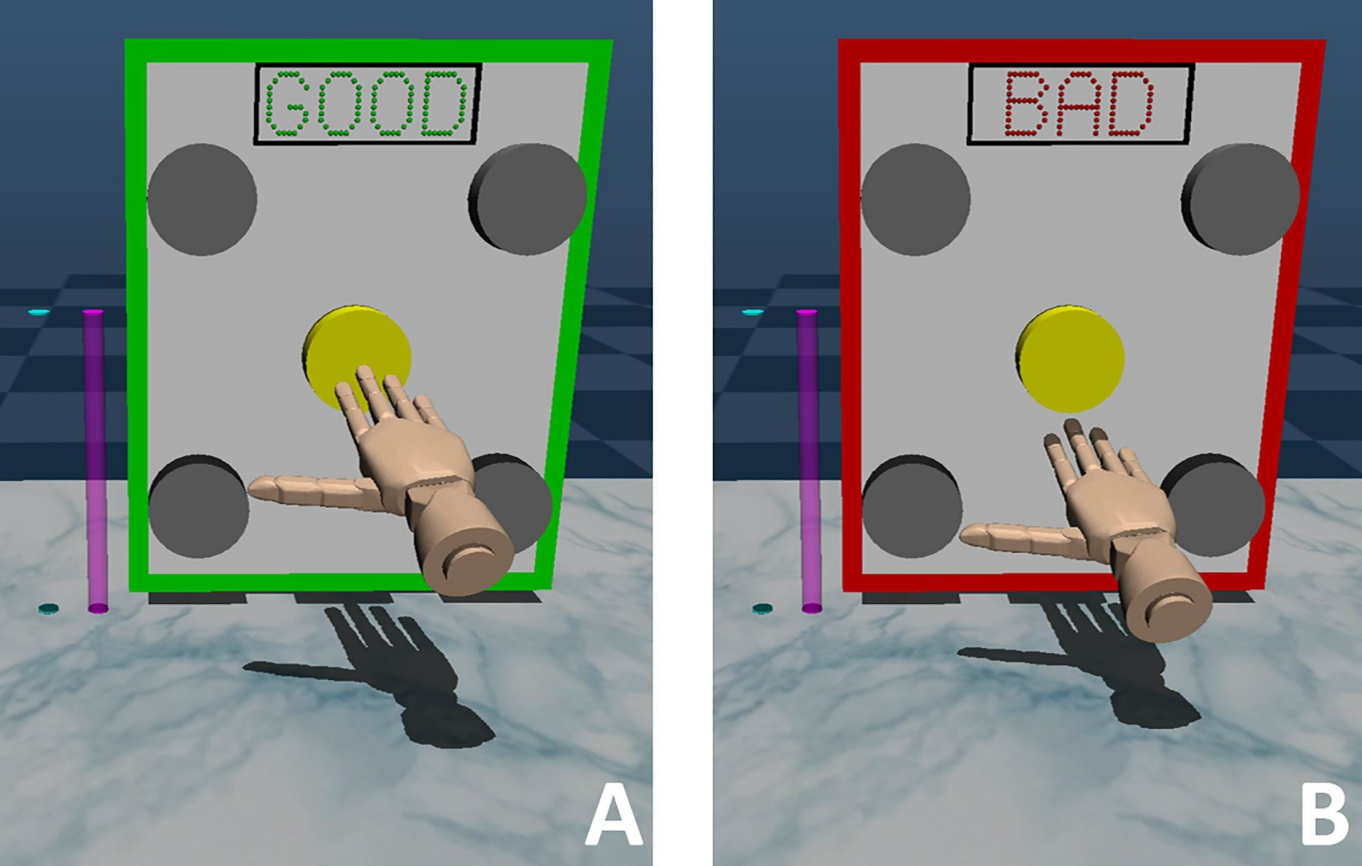
Example trials of message feedback provided upon touch of virtual hand on highlighted (yellow) target. **A)** ‘GOOD’ message interpreted as positive feedback. **B)** ‘BAD’ message interpreted as negative feedback. *Positive feedback* training sessions initially prescribed a ‘GOOD’ for 80% of trials. *Punishment feedback* training sessions initially prescribed a ‘GOOD’ message for 20% of trials. All other trials provided ‘BAD’ message.

*Virtual environment*. The virtual environment consisted of the following: (1) virtual hand that moved with the subject’s real hand, (2) target panel with white background embedded with five grey circle targets to touch, (3) rectangular box at top of panel that displayed positive (‘GOOD’) or negative (‘BAD’) feedback message following target touch (**Fig 3**), and (5) two vertical projection cylinders left of the panel. At the start of each trial, the headset displayed the virtual hand positioned coincident with the subject’s real hand relative to their head to facilitate sense of embodiment [38]. Position changes of the marker cluster on the real hand had 1:1 matching with position changes of the hand in the virtual environment. The first cylinder (cyan) moved downward at the start of each trial as a countdown to begin reach. The second cylinder (pink) moved upward for three seconds following countdown completion of the first cylinder and served to pace timing of touch.

**Fig 4:**
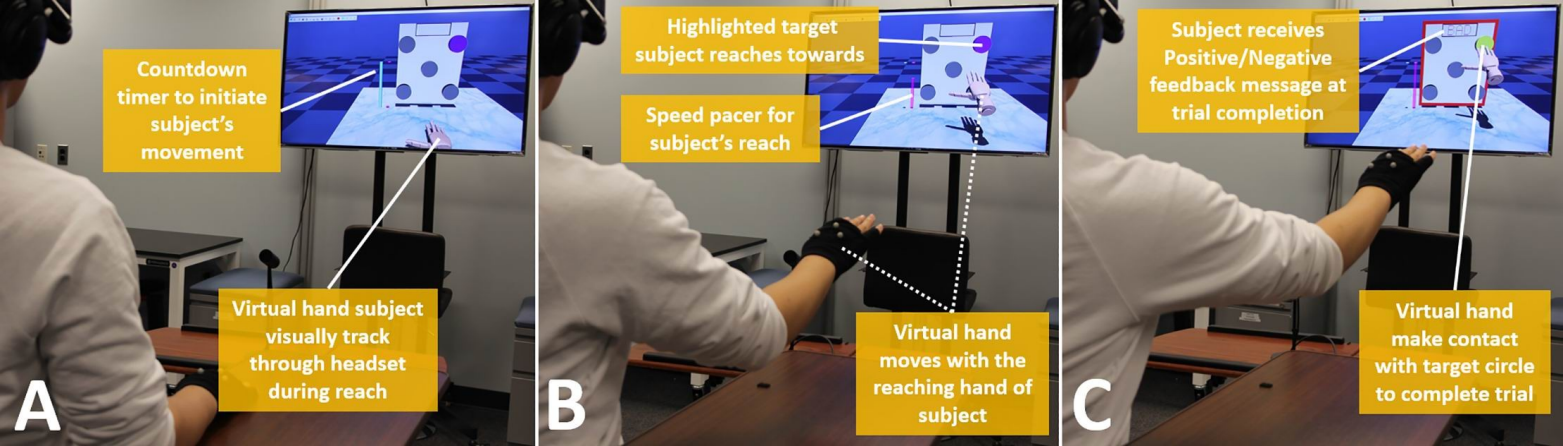
Virtual Reality environment–**A)** Subject hand and virtual hand initial position with virtual timer before subject’s movement, **B)** Subject hand and virtual hand reaching for the targeting circle against speed tracker, **C)** Positive/Negative visual feedback provided with virtual hand make contact with target circle.

*Task protocol*. At the start of each trial, one of the five target circles changed colors from grey to blue to inform the subject which target to reach towards and touch. The subject was instructed to reach towards the blue target after the countdown cylinder completed its movement (**Fig 4**). While reaching, the subject would keep in mind *three performance objectives*: (1) minimize reaching path length, (2) time touch contact at 3 seconds of reach, (3) accurately touch the center of the target. Subjects were told to prioritize the objective of minimizing reach path length while adhering to the contact objectives as best as possible. Touch contact was indicated to the subject when the designated target turned yellow. This event also marked the end of further movement for that trial. Touch contact between the virtual hand and the target object was flagged through a contact-detect function available in the physics engine of this virtual environment. For training trials, the subject also received positive or negative feedback in the form of a ‘GOOD’ or ‘BAD’ message immediately upon contact; otherwise for non-training trials, no message was posted. The subject was previously told a ‘GOOD’ or ‘BAD’ signified above or below average performance, respectively, compared to previous subjects. A beep was sounded to the headphones at some variable time-interval after touch contact. For agency assessment, the subject estimated the time-interval in denominations of 100 msec between 100 and 1000 msec. The actual time-intervals were randomized within each testing block and Gaussian-distributed with center at 500 msec.

#### Experimental testing blocks

**Fig 5:**
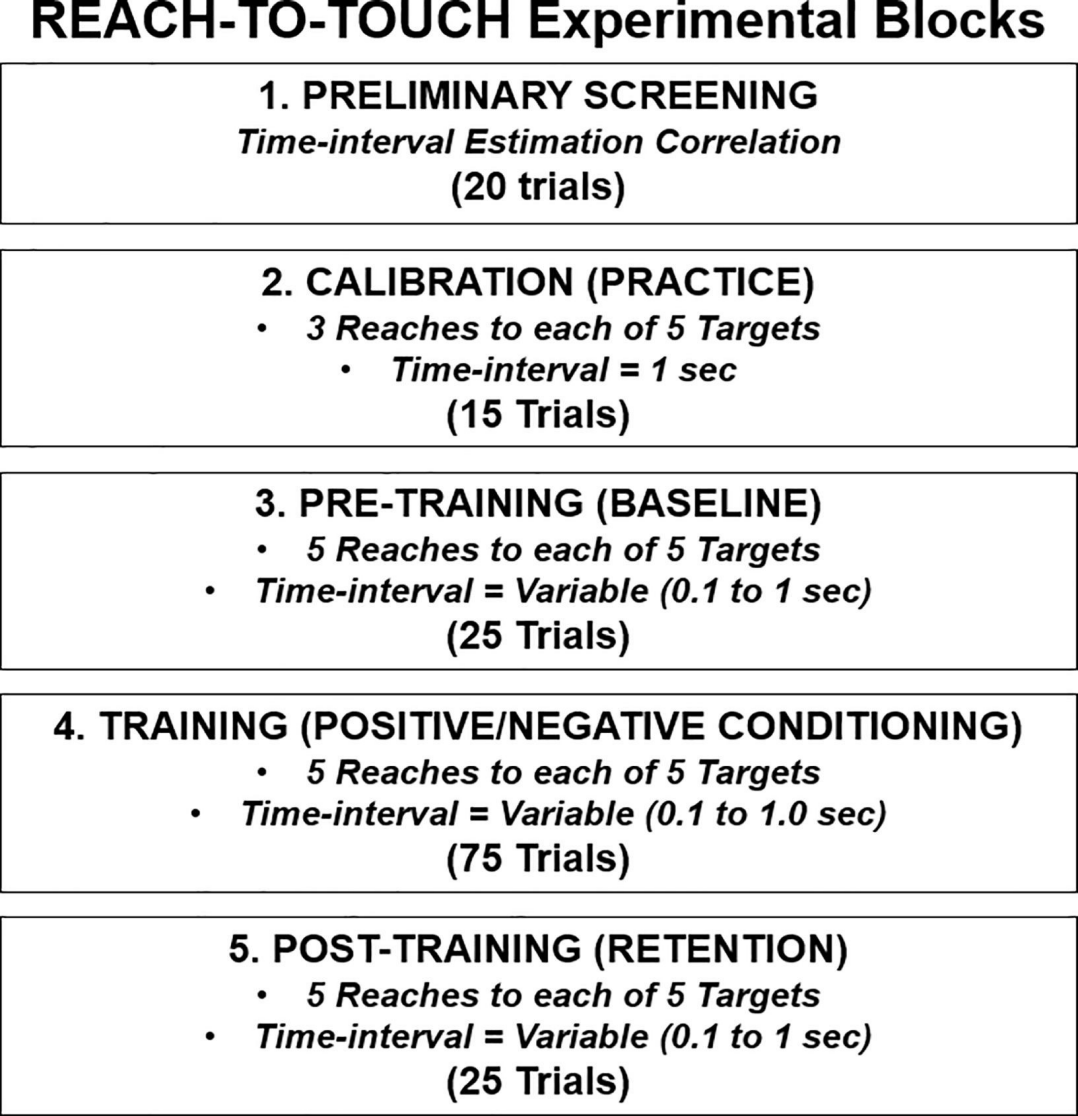
Experimental protocol included five major testing blocks: (1) Screen participants for sufficient ability to estimate time-intervals, (2) Allow subjects to practice virtual reaching and calibrate internal reference of 1 second time-interval, (3) Establish baseline performance for virtual reach-to-touch task, (4) Provide training that conditions subject to either reward or punishment feedback, (5) Observe short-term retention effects of positive or negative conditioning on performance of reach-to-touch for subsequent comparison to baseline.

The experimental protocol consists of 5 major testing blocks (**Fig 5**). To minimize fatigue, a rest period of 5 to 10 minutes was provided after each block. The rest period included removal of headset and headphones. The testing blocks are described as follows:

1. PRELIMINARY SCREENING: Prior to participation in the full experiment protocol, subjects performed 20 reach-to-touch trials to assess prospective subject ability to estimate time-intervals. Only subjects whose estimates had correlation greater than 0.5 with actual time-intervals were retained for further testing. This exclusion ensured fundamental ability to discriminate short from long time-intervals and not randomly ‘guess’.
2. CALIBRATION: Subjects performed 15 practice trials (3 trials to each of 5 targets) with 1 second time-interval between touch and audio beep. These trials accommodated the subject to all targets and provided an internal reference for a 1 second time-interval.
3. PRE-TRAINING: This block started the experiment proper. The subject performed 25 trials (5 reach-to-touch to each of the 5 targets, randomly presented) with no message feedback. Performance during this block served as the ‘baseline’ for each subject.
4. TRAINING: In this block, the subject performed 75 trials with positive/negative feedback after each trial to ‘condition’ each subject. Without the subject’s knowledge, each subject was pre-determined to belong to either the ‘positive’ or ‘negative’ conditioning group. Each subject was initially prescribed to receive a disproportionate (80% of trials) preponderance of ‘GOOD’ or ‘BAD’ messages for positive or negative feedback training, respectively. All training trials provided either a ‘GOOD’ or ‘BAD’ message. Exception to the predetermined positive/negative message for each trial was if a subject notably over or under performed (see ‘Trial Override’). To minimize fatigue, the 75 trials were presented as three sub-blocks of 25 trials with 2–3 minutes of rest between blocks. Prior to the 25-trial block, the subject was allowed up to three practice trials for re-accommodation.
5. POST-TRAINING: This final block included the same presentation of 25 trials as in pre-training with the same target order and no message feedback. Performance and agency measured during this block served as comparison to baseline for each subject and to assess the retention effects from training.

#### Trial override

Override of prescribed feedback was done for training trials when subjects performed exceptionally well or poorly so that predetermined feedback did not appear paradoxical. This contingency ensured subjects were not confused by clearly erroneous feedback about performance. The standards for exceptional performance was based on each subject’s own performance during the pre-training (baseline) block. The override conditions, based on heuristic observations from pilot experiments were as follows:

1. Subject was guaranteed to receive ‘GOOD’ or ‘BAD’ message if path length was > 3 standard deviations shorter or longer, respectively, than the mean from baseline.
2. Subject was guaranteed to receive ‘BAD’ if time to touch contact was < 1.5 sec or > 4 sec following the countdown to initiate reach.
3. Subject was guaranteed to receive ‘GOOD’ or ‘BAD’ message if contact accuracy was > 3 standard deviations better or worse, respectively, than the mean from baseline.

The override condition was the only instance when feedback was necessarily indicative of true performance for a given trial, and it was expected overrides would only occur occasionally.

#### MHLC surveys

Subjects were asked to complete a survey of multidimensional health locus of control (*MHLC*) [54] to identify underlying personal beliefs [55]. MHLC responses indicate whether one believes their health status is under their own control or under external forces [56]. External forces include other people, circumstances, or “a higher power”. The MHLC survey included 54 questions with three generalized categorical factors on health outcome: internality, power, and chance. These factors are not mutually exclusive but can vary independently. *Internality* indicates belief in one’s own control, *power* indicates belief in the effect of external factors, and *chance* indicates belief in random influences. Subjects were instructed to answer each question on a 6-point Likert scale as to what extent they agreed with a presented statement. Subjects were informed that there were no right or wrong answers. Subjects completed the survey at least one day either before or after the VR protocol depending on their availability. A total of 18 individuals took the survey as four subjects were non-respondent following the VR protocol. Subject recruitment was independent of potential MHLC responses and there was no guarantee of attaining a broad range in survey scores.

### Data analysis

#### Metrics

1. Agency–Positive measurements for agency were indicated by underestimation of actual time-intervals. Underestimation suggested relative compression in perception of time-interval (Δt) between action (touch) and consequence (sound beep). Agency was defined as Δt_true_—Δt_estimated_, so more positive value indicated greater agency.
2. Normalized path length–Path length data was normalized since there were variable reaching distances across the targets. Each 3-D reaching pathlength was normalized by the minimum path length (straight line) between the tip of the middle finger of the virtual hand at the starting position and the center of the target for that trial. Explicit computation of normalized path length is as follows:
  $$\bar{P}=\frac{1}{P}∙\sum \sqrt{{\left(x_{i}-x_{i-1}\right)}^{2}+({y_{i}-y_{i-1})}^{2}+{\left(z_{i}-z_{i-1}\right)}^{2}}$$ <Eq 1>
  where $\bar{P}$ is the normalized path length; *P* is the minimum path length to a target; *x,y,z* are individual position coordinates of the hand marker cluster; and, *i* is the sample-time index summed across the reach-to-touch trajectory. We assumed sampling rate was sufficiently high (120 Hz) to assume linear increments in path length between sampled time points. The minimum path length to the center target was 30 cm. The minimum path length for either top target was 42 cm. The minimum path length for either bottom target was 20 cm.
3. Contact timing–The target time at which to make contact was 3 seconds (i.e., 3000 msec) after the countdown to begin reach. The actual time to make contact was logged and analyzed across subjects. Based on pilot experiments [51], we chose not to interpret this metric as an ‘error’ to the target time since subjects were highly accurate (<200 msec error) at baseline. Furthermore, high accuracy persisted regardless of reward or punishment feedback.
4. Contact location error–Higher contact accuracy was measured as smaller absolute error (3D distance) between the actual location of contact and the center position of each target. Although the target object itself represents a 2D plane, the explicit computation of location error (*E*) in 3D (*x,y,z* position within global coordinate system of virtual environment) is as follows:
  $$E=\sqrt{{\left(x_{t}-x_{c}\right)}^{2}+({y_{t}-y_{c})}^{2}+({z_{t}-z_{c})}^{2}}$$ <Eq 2>
  where *t* and *c* is the position index for the target center and actual contact, respectively.

#### Statistics

Paired t-tests were used for to evaluate significant differences (p <0 .05) in agency and performance metrics between reward and punishment groups after training. A linear regression was used to evaluate significant progressive changes in performance and agency across trials during training. A linear regression was also used to relate each MHLC category (‘Internality’, ‘Chance’, and ‘Powerful others’) to agency across subjects.

## 3 Results

**Fig 6:**
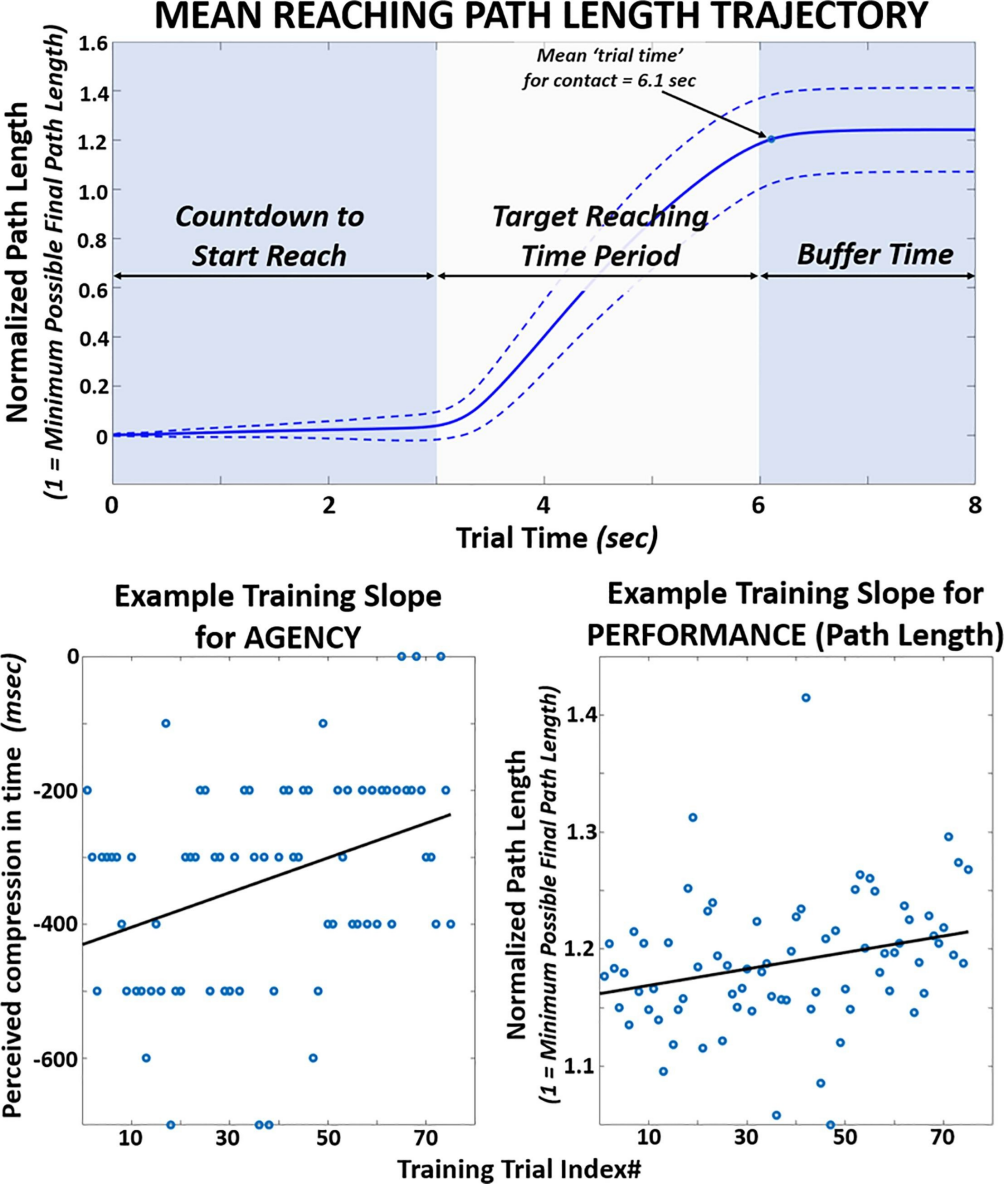
Typical performance and agency observed during reach-to-touch task. TOP: Mean accumulation of path length over time for reaching trajectory during baseline. Path length accumulation plotted against trial time (0 to 8 seconds). All pathlength accumulation data normalized (divided) by the minimum ‘final’ pathlength for each respective trial. This minimum ‘final’ pathlength is the linear distance between initial hand position and center of target for that trial. BOTTOM: Example slope fits for agency and performance data during 75-trial training session for one subject receiving disproportionate positive feedback.

Normality for all agency and performance data was verified using the Kolmogorov-Smirnov test to validate the use of parametric statistical tests. **Fig 6 (TOP)** shows the average path length reaching trajectory across all 22 subjects. Also, **Fig 6 (BOTTOM)** shows sample agency and performance (path length) data collected for one subject during the 75-trial training session with reward. The fitted line from linear regression is superimposed onto each data set. This ‘training slope’ from the linear fit was logged for each subject to compare progressive changes during training between positive versus negative feedback.

**Fig 7:**
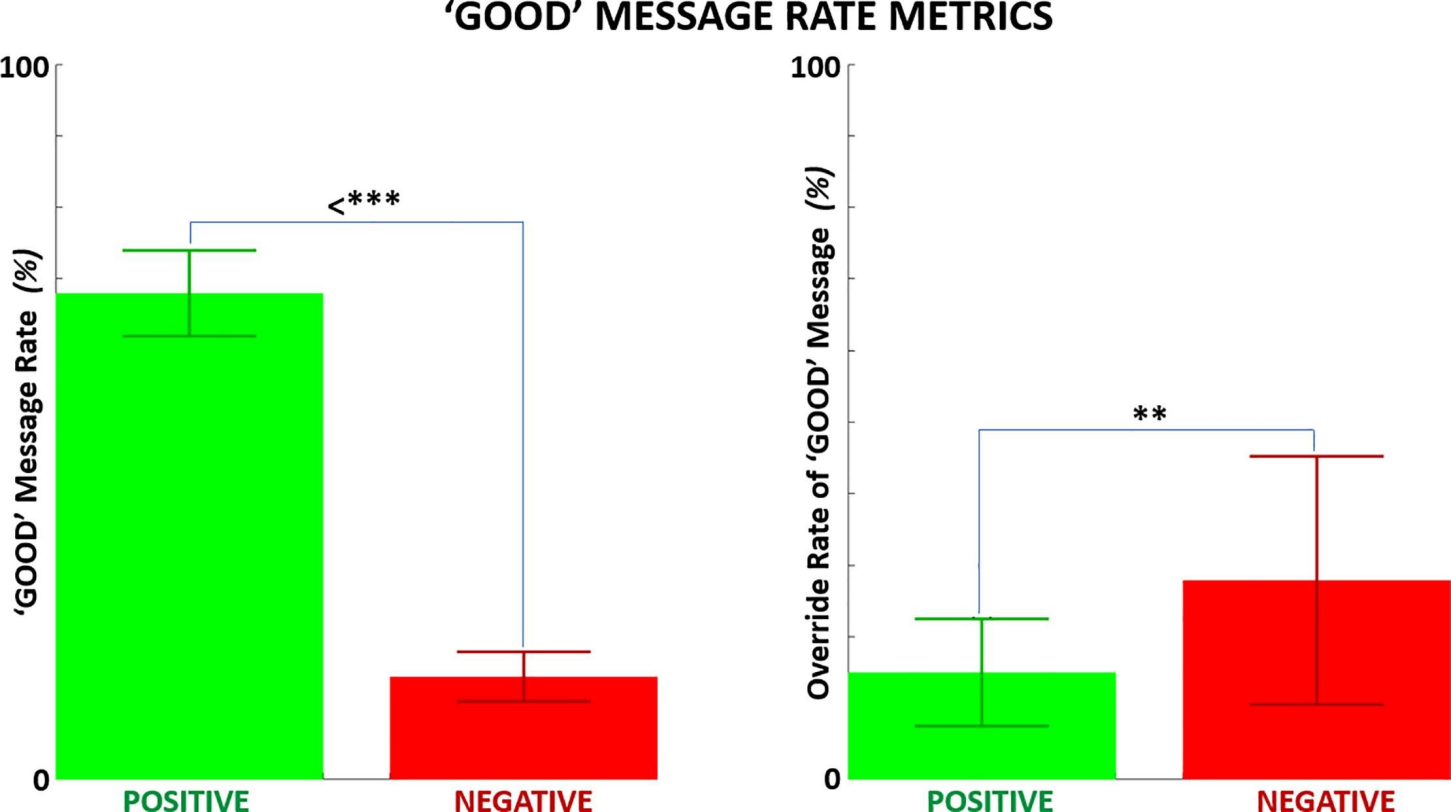
Mean ‘GOOD’ message rate metrics shown for disproportionate positive versus negative feedback training sessions. LEFT*—*Net rate of ‘GOOD’ message during disproportionate positive versus negative feedback. Participants initially prescribed to receive ‘GOOD’ message 80% and 20% of trials during disproportionate positive and negative feedback, respectively. Deviations from initially expected rates due to select trial overrides generated by outlier performance. RIGHT–The net override rate of ‘GOOD’ message trials converted to ‘BAD’ message due to outlier poor performance shown for both feedback groups.

While each subject was initially prescribed to receive reward (80% ‘GOOD’ feedback) or punishment (80% ‘BAD’ feedback) training, the effective rate of feedback differed based on trial overrides when subjects perform exceptionally well or poor. **Fig 7 (LEFT)** and **Table 1** show that the average effective rate of ‘GOOD’ messages for the positive feedback (PF) group was 68% and for the negative feedback (NF) group was 14%. Given a reduction from the prescribed rates of 80% and 20%, there was greater net override of positive messaging due to select poor performance for both PF and NF groups. **Fig 7 (RIGHT)** shows that the override rate of positive messaging was greater for the NF group (28%) than the PF group (15%). Even in the presence of override, the effective dosage of positive messaging was significantly greater (54%, p < 3E-14) for the PF group than the NF group and with very large effect size (Cohen’s D = 10.94) as intended for this protocol. The override rate during NF was significantly greater (p = 0.002) with large effect (Cohen’s D = 0.964) compared to PF.

**Table 1:** Comparing positive feedback rate, including override, during positive feedback (PF) and negative feedback (NF) training.

METRIC	*PF*	*NF*	p-val	Cohen’s D	t-statistic
*Net Positive Feedback Rate (%)*	68 ± 6	14 ± 4	**2.72E-14**	10.94	62.4
*Override Rate of Positive Feedback (%)*	15 ± 8	28 ± 17	**0.002**	0.964	4.1

Significant post hoc p-values (< 0.05) bolded

**Fig 8:**
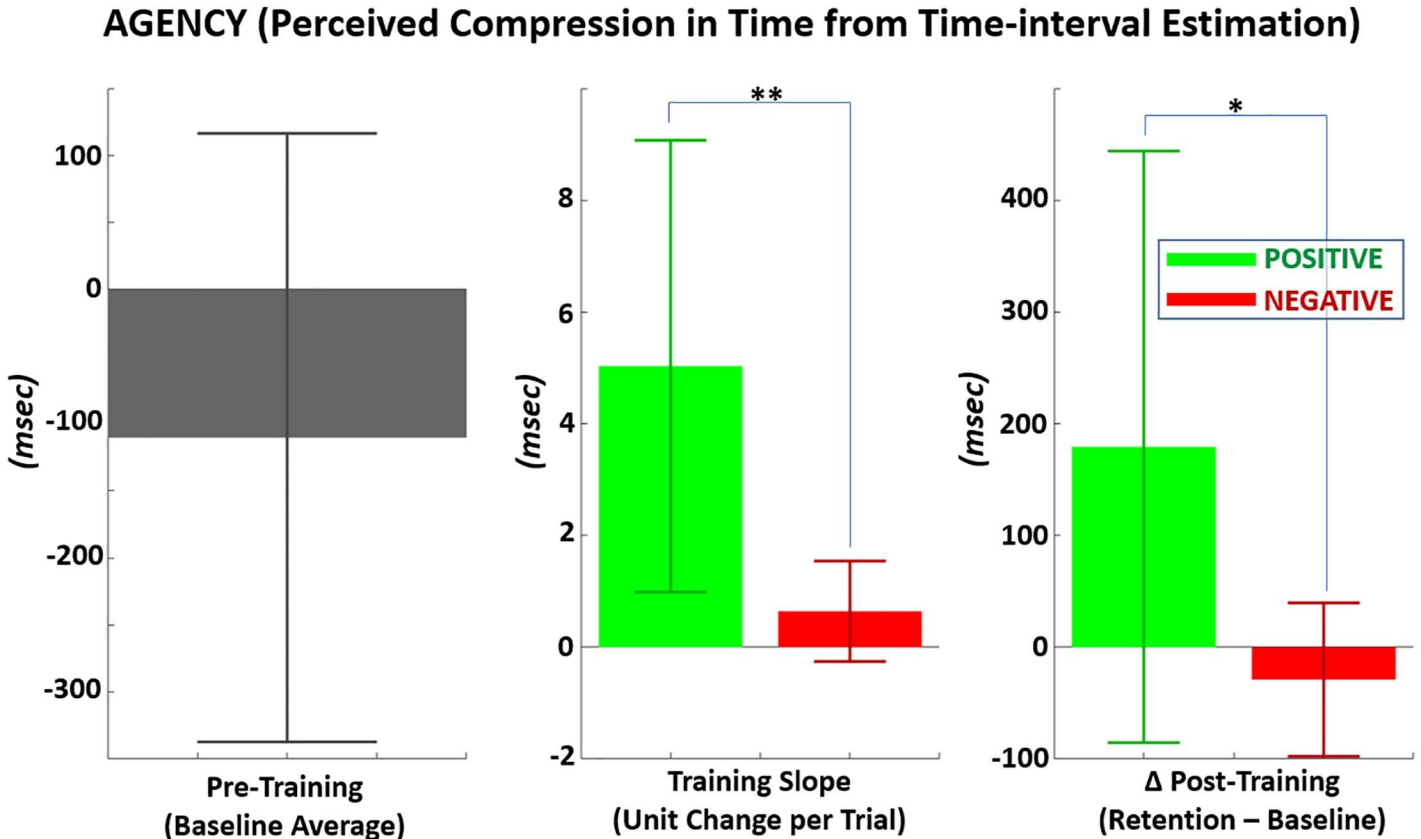
Agency measured as compression in perceived time-interval between action (target touch) and proceeding sensory consequence (auditory beep). LEFT*–*Mean agency prior to training (baseline), MIDDLE*–*Per trial change in agency during training with positive versus negative feedback, RIGHT*–*Mean change in agency from baseline following training with positive versus negative.

**Fig 8** and **Table 2** show the following results for agency: 1) at baseline, 2) progressive rate of change during training, and 3) change from baseline at retention (post-training). The mean agency at baseline was -110 ± 227 msec. Negative agency indicates time-intervals were perceived to be longer than actual values on average. Across trials for both reward and punishment training, the fitted linear regression slope of agency was positive. This result suggests agency progressively increased during training. The training slope during PF (5.03 ± 4.04 msec/trial) was significantly greater (p < 0.01) than NF (0.64 ± 0.90 msec/trial) with large effect size (Cohen’s D = 1.50). Similarly, there was a greater positive increase in agency post-training with PF (179 ± 265 msec) compared to NF (-29 ± 69 msec). This greater increase in agency with PF was significant (p = 0.036) with large effect size (Cohen’s D = 1.08). A one-sample t-test also confirmed that the positive change in agency with PF was significantly different from zero for both training slope (p < 0.05) and post-training change from baseline (p < 0.05). Significance from zero was not observed for NF (p > 0.05) for either training slope or post-training change in agency.

**Table 2:** A. Training slope in agency and performance metrics for positive feedback (PF) versus negative feedback (NF) training. **B**. Mean change in agency and performance metrics from baseline after PF versus NF training.

**A**. Training slope in agency and performance metrics for positive feedback (PF) versus negative feedback (NF) training							
METRIC	*Baseline value*	*PF Slope (change/trial)*		*NF Slope (change/trial)*	p-val	Cohen’s D	t-statistic
*Agency (msec)*	-110 ± 227	5.03 ± 4.04		0.64 ± 0.90	**.0058**	1.50	3.18
*Performance–Path length (cm)*	34 ± 3.6	0.034 ± 0.039		0.0026 ± 0.130	**0.036**	1.08	2.28
*Performance–Contact Timing (msec)*	3107 ± 474	-3.77 ± 4.16		-0.33 ± 0.27	**0.035**	1.17	2.35
*Performance–Contact Location Error (cm)*	2.28 ± 2.79	-0.02 ± 0.06		-5E-4 ± 1E-3	0.356	0.448	0.95
**B**. Mean change in agency and performance metrics from baseline after PF versus NF training							
METRIC		Δ *post-PF*		Δ *post-NF*	p-val	Cohen’s D	t-statistic
*Agency (msec)*		179 ± 265	-29 ± 69	**0.036**	1.08		2.28
*Performance–Path length (cm)*		-1.76 ± 1.72	-0.179 ± 0.60	**0.0381**	1.23		2.31
*Performance–Contact Timing (msec)*		-212 ± 244	-36 ± 45	**0.049**	1.01		2.13
*Performance–Contact Location Error (cm)*		-0.23 ± 0.66	0.001 ± 0.01	0.36	0.51		0.95

Significant post hoc p-values (< 0.05) bolded

**Fig 9:**
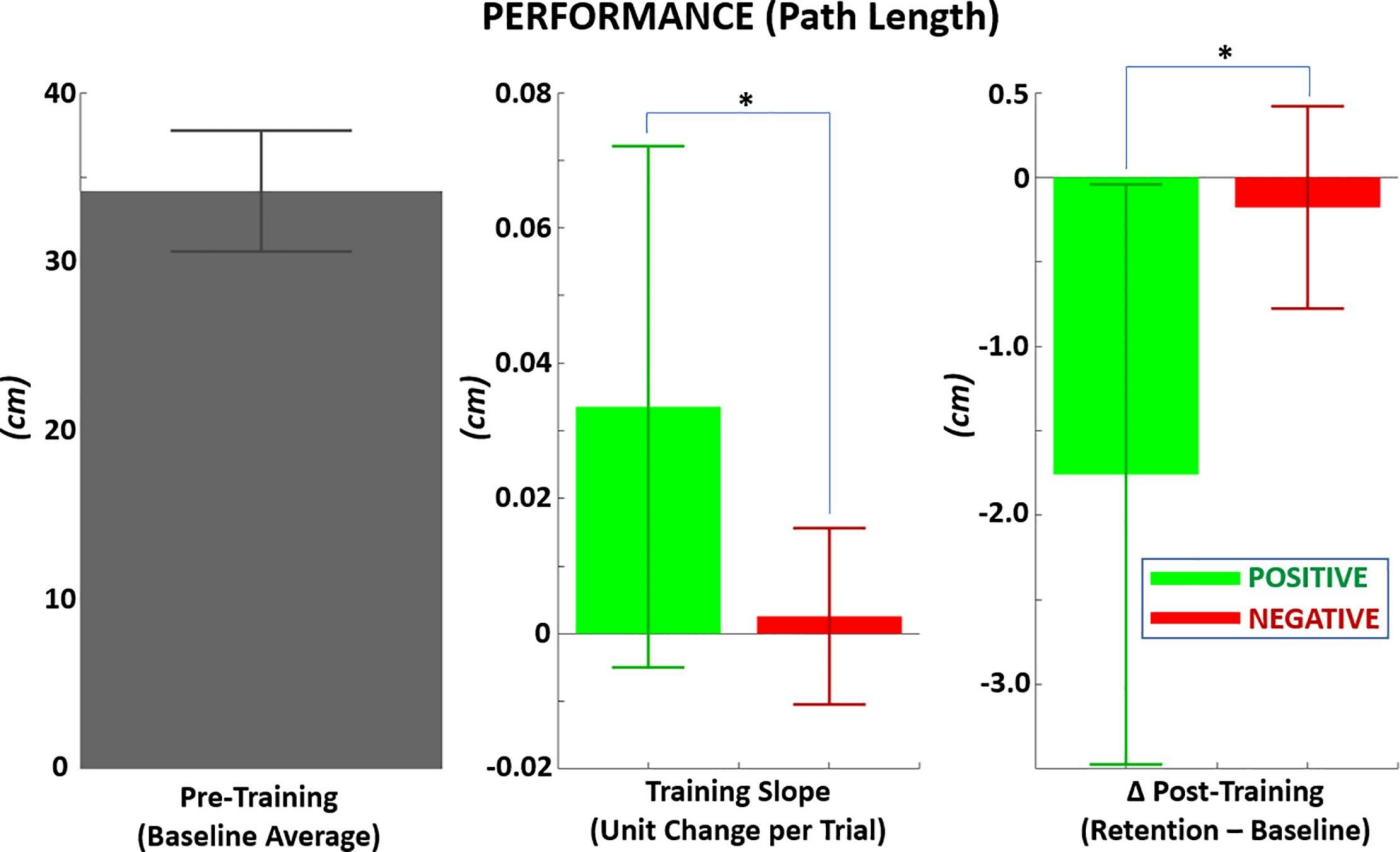
Performance metric of path length in hand trajectory measured during reach-to-touch task. LEFT*–*Mean path length prior to training (baseline), MIDDLE*–*Per trial change in path length during training with positive versus negative feedback, RIGHT*–*Mean change in path length from baseline following training with positive versus negative feedback. all y-axis data in ‘cm’ after normalized path length data re-multiplied by 30 cm, the minimum path length to the center target.

**Fig 9** and **Table 2** show results for the primary performance metric of path length: 1) at baseline, 2) progressive rate of change during training, and 3) change from baseline at retention (post-training). All path length data for each trial was normalized by the minimum path length for that trial, and then re-multiplied by 30 cm (the minimum path length to the center target). The resulting mean path length at baseline across all trials and targets was 34 ± 3.6 cm. Shorter path length values indicated greater performance given the objective to minimize path length. For both PF and NF training trials, the fitted linear regression slope of path length was positive. This result suggests there was progressive increase in path length during training. The training slope during reward (0.034 ± 0.039 cm/trial) was significantly greater (p < 0.05) than punishment (0.0026 ± 0.130 cm/trial) with large effect size (Cohen’s D = 1.08). Despite progressive increase in path length during training, there were post-training reductions in path length for both PF (-1.76 ± 1.72 cm) and NF (-0.179 ± 0.60 cm). The retention reduction in path length post-reward was significantly greater (p < 0.05) than post-punishment with large effect size (Cohen’s D = 1.23). As with agency, one-sample t-test showed significant change in performance with PF for both the training slope (p < 0.05) and post-training change from baseline (p < 0.05). Also similar to agency, significant difference from zero was not observed with NF (p > 0.05) for either the training slope or post-training change in performance.

**Fig 10:**
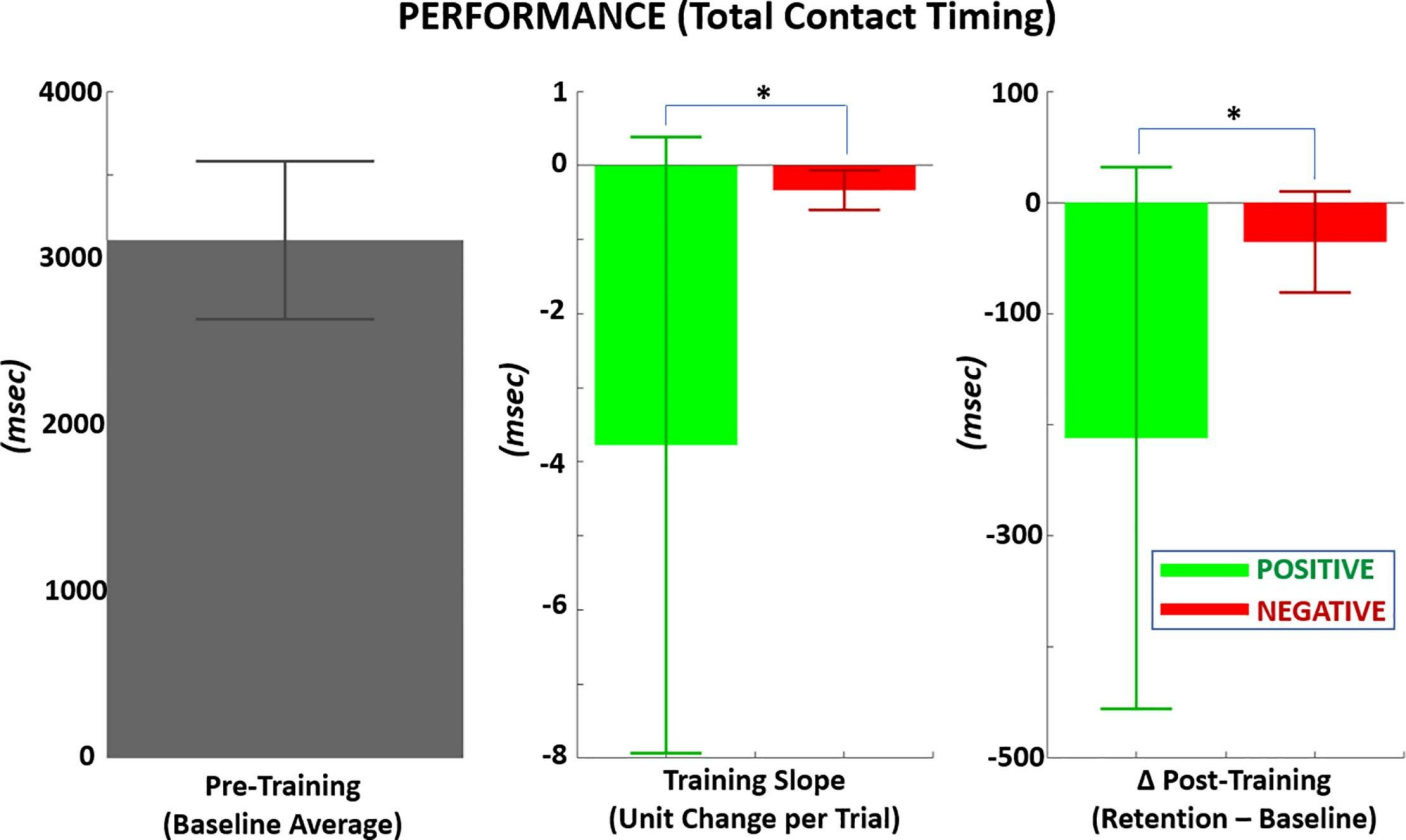
Performance metric of mean time to contact touch a target with the virtual hand during reach-to-touch task. The target touch time was 3000 msec. LEFT*–*Mean contact timing prior to training (baseline), MIDDLE*–*Per trial change in contact timing during training with reward versus punishment, RIGHT*–*Mean change in contact timing from baseline following training with positive versus negative feedback.

**Fig 10** shows that the average contact time at baseline was ~3107 msec following the cue to start reach (at trial time = 3 seconds). The desired contact time to match the speed pacer was 3000 msec. Training with PF reduced contact time across training (-3.77 ± 4.16 msec/trial) and resulted in a post-training reduction from baseline (-212 ± 244 msec). Both of the reductions in contact timing with PF were significantly greater than with NF (p < 0.05) and with large effect size (Cohen’s D > 1). However, there was no significant difference (p > 0.05) between PF and NF in timing error (or difference) from the ideal contact time of 3000 msec. From the baseline mean, the ideal change in contact time would be a reduction of ~107 msec.

**Fig 11:**
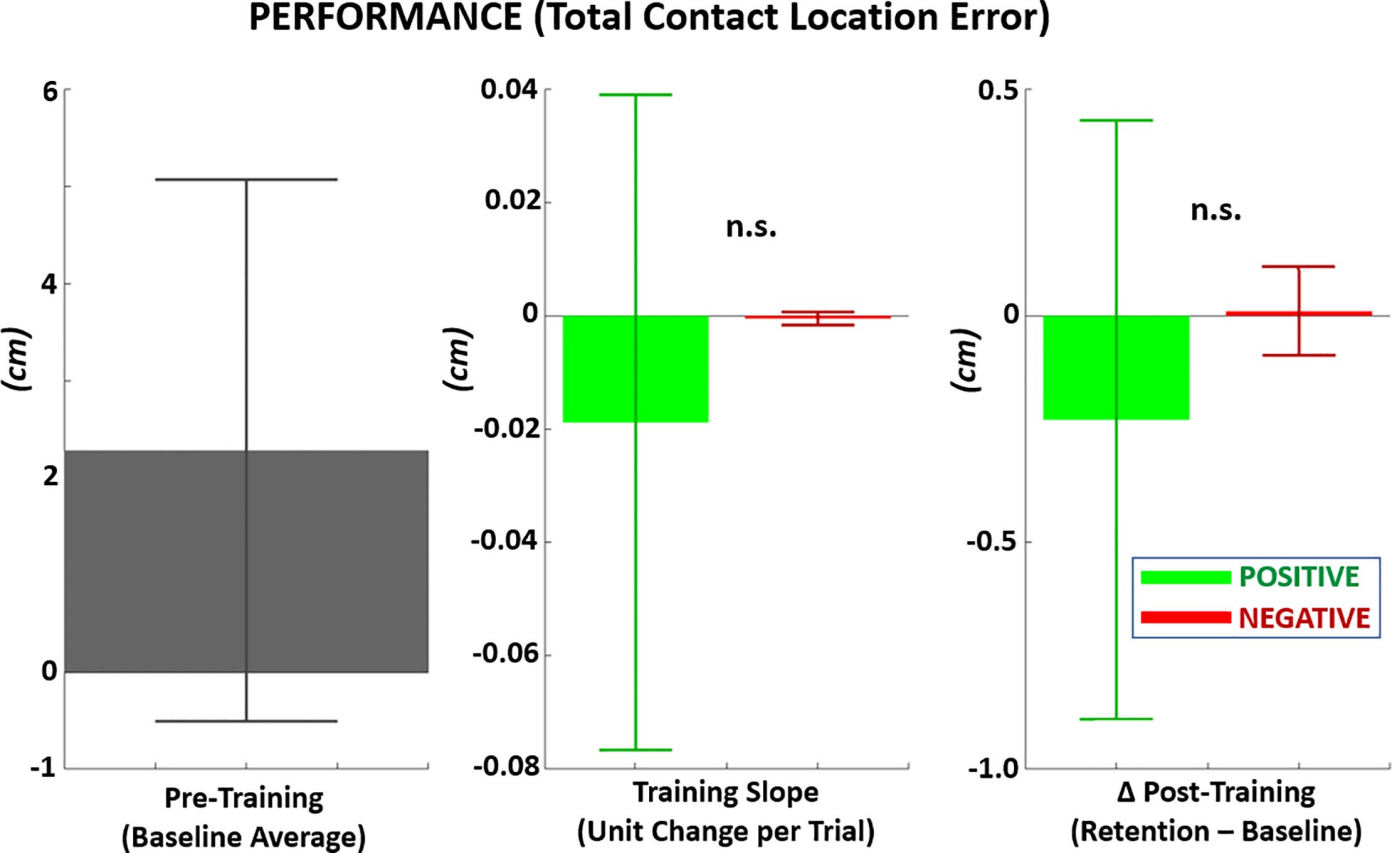
Performance metric of contact accuracy measured as distance error between location of contact touch of hand to center of target during reach-to-touch task. LEFT*–*Mean contact error prior to training (baseline), MIDDLE*–*Per trial change in contact error during training with positive versus negative feedback, RIGHT*–*Mean change in contact error from baseline following training with positive versus negative feedback.

**Fig 11** shows that the average contact location error to the center of the touch-target was 2.28 ± 2.79 cm. While PF produced net reductions in error for the training slope and the post-training change from baseline, these reductions were not significantly different compared to NF (p > 0.05). Furthermore, a one-sample t-test power analysis at 80% suggested a sample size of 76 would be necessary to demonstrate a non-trivial reduction with reward (i.e., reduction significantly different from zero).

**Fig 12:**
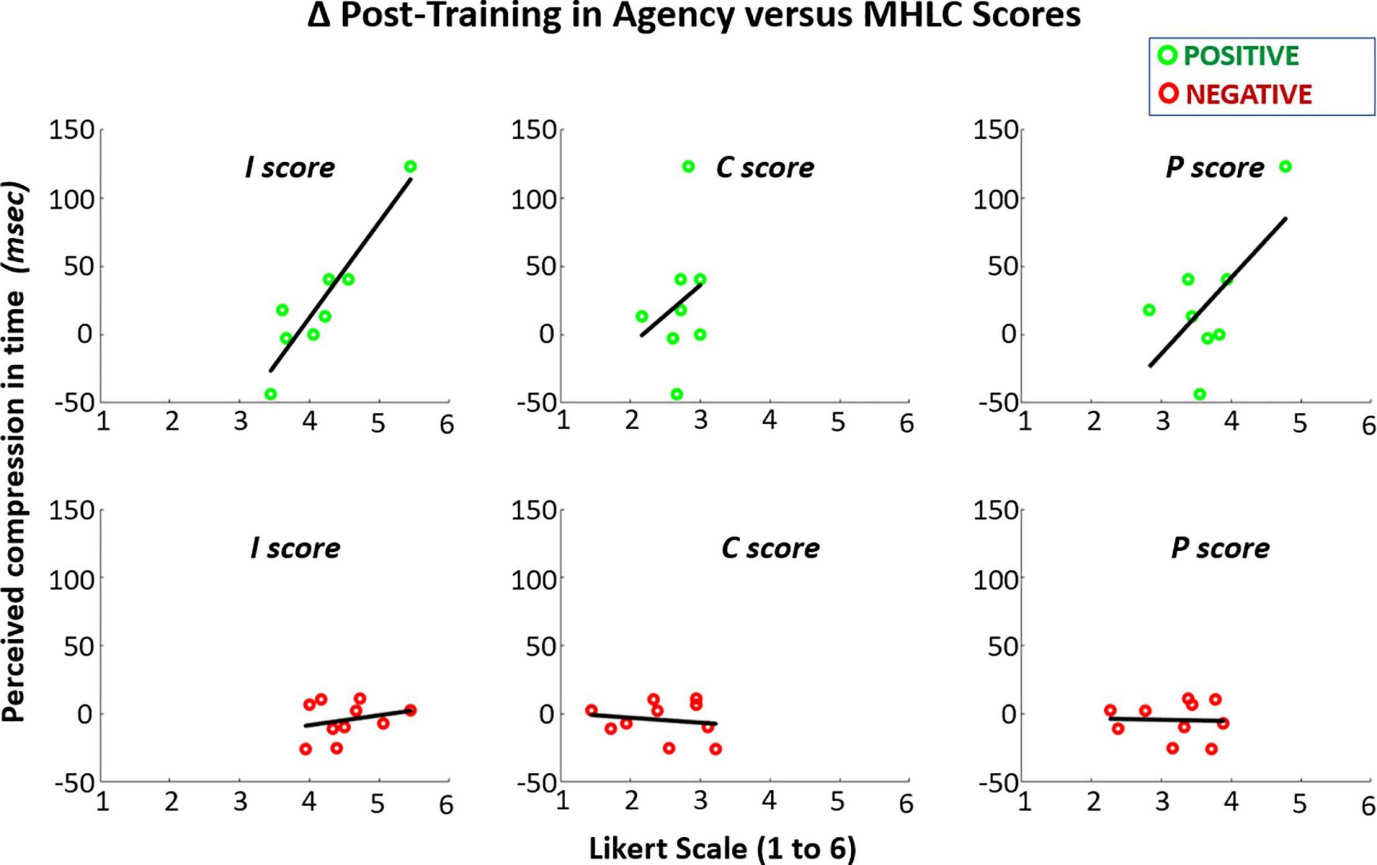
Average change in agency after either reward or punishment training is plotted against scores from MHLC (multi-dimensional) health locus of control survey for internality (*I*), chance (*C*), and power of others (*P*).

A total of 8 subjects took the MHLC survey and participated in PF training while 10 subjects took the survey and participated in NF training. The scores for average internality (I), chance (C), and power of others (P) scores from the MHLC surveys are shown in **Table 3**. In **Fig 12**, the scores are shown against mean post-training change in agency for individual subjects. No significant difference (p > 0.05) was observed between PF and NF for any of the three scores. Only the linear regression slope between internality and agency for reward subjects was found to be significantly different from zero (slope = 0.70 msec/unit-score, p < 0.001).

**Table 3:** MHLC scores and correlation to post-training change in agency for positive feedback (PF) and negative feedback (NF).

			Linear Regression Results		
METRIC	*Mean Score (Likert Scale 1 to 6)*	*Mean Agency (msec)*	*Slope Value (msec / unit-score)*	p-val	t-statistic
*I Score (PF)*	4.16 ± 0.64	235 ± 48	0.70	**8E-4**	6.22
*C Score (PF)*	2.71 ± 0.26	235 ± 48	0.44	0.57	0.61
*P score (PF)*	3.68 ± 0.56	235 ± 48	0.56	0.09	2.05
*I Score (NF)*	4.52 ± 0.47	-45 ± 135	0.07	0.48	0.74
*C Score (NF)*	2.46 ± 0.61	-45 ± 135	0.04	0.65	0.47
*P score (NF)*	3.22 ± 0.57	-45 ± 135	0.01	0.91	0.11

Significant post hoc p-values (< 0.05) bolded

## 4 Discussion

The primary finding of this study is that conditioning with disproportionate simple positive feedback, compared to negative feedback, produced significant increases in both agency and reaching performance with a virtual hand. Feedback in this study is characterized as simple and binary due to the provision of either a ‘GOOD’ (positive) or ‘BAD’ (negative) message with each trial. While performance-based reward alone is known to accelerate motor learning [57], the concurrent effect of general positive feedback, regardless of performance, on the person’s perception of movement control has not been well established. There has been demonstration of dissociative effects of reward and punishment on motor learning. Punishment can enhance motor learning with visuomotor rotations or be associated with faster re-adaptation [58]. However, the perceptual and performance effects of cognitive satisfaction from positive or negative feedback independent of performance during training has not been isolated or investigated. Discriminating the relative effects of positive and negative feedback on cognitive factors such as motivation or perception of performance may inform design of better rehabilitation protocols. Commitment to physical therapy is key to achieving functional gains, and rehabilitation methods that optimize perception can be invaluable. In our study, we specifically observed the effects of positive and negative feedback on VR reach-to-touch, a potential platform for customized neuromotor rehabilitation.

Elements to enhance neuromotor rehabilitation include task feedback, task complexity, and amount of practice [17, 59]. Our study examined binary feedback for a simple virtual reaching task with limited practice. VR environments readily allow for systematic changes in feedback, simulated task complexity, and customized aspects of entertainment [60, 61]. However, it remains unclear what is the benefit of controlled-dosage VR and the specific ingredients of VR that actively produce desired outcomes [62]. Thus, identification of optimal VR design features is critical to accelerate rehabilitation performance. These design features include how feedback is provided, and what are the expected effects during and after training.

In this study, training trials were initially prescribed at a rate of 80% positive messaging with the positive feedback and 20% with the negative feedback group. Pilot testing indicated that the 80% threshold, with expected level of overrides, was conceivably realistic to each subject without eliciting apparent frustration or carelessness. With overrides, the effective provision of positive messaging during positive feedback training was 68% on average, but still significantly greater (p < 0.0001) with large effect compared to negative feedback training (14% positive messaging). It was assumed the positive and negative feedback cohorts were preserved as designed given the significantly greater positive messaging (+54%) with the PF group. An interesting finding, not intrinsic to our central hypotheses, was that the override rate of positive messaging was greater during NF training. This suggested that negative feedback induced a higher frequency of exceptionally (outlier) poor performance trials during training.

The primary hypothesis of this study was that positive feedback would increase agency and performance. We assessed this hypothesis from post-training changes (relative to baseline) and progressive changes (slope over trials) during training. Our secondary hypothesis was that internal beliefs, as measured by MHLC survey responses, show dependence on agency based on punishment or reward conditioning. The main findings for these hypotheses are discussed further as follows:

### 4.1 Positive feedback training increased agency and reaching performance from baseline compared to punishment

Extrinsically motivated states that facilitate goal-oriented performance can be identified from Reward Positivity, an event-related potential component following positive appraisal [63, 64]. The suppression of beta activity that occurs with more motivated states also leads to enhanced preparation for actions, manifested by faster reaction times [65, 66]. Furthermore, our finding of improved reaching performance with positive feedback substantiates previous literature citing motor memory forged from dopaminergic experiences [6]. Sense of agency is also known to be associated with dopaminergic pathways in studies involving priming and disease [67, 68]. To the best of our knowledge, this is the first study to relate functional performance and agency with implications for VR neuromotor rehabilitation. We observed that positive feedback substantially increased agency and reaching performance compared to negative feedback. This is an importance distinction since previous work has shown that punishment can enhance motor learning and has dissociable effects from reward [47, 58, 69].

Our findings suggest that performance feedback in VR, whether informative or strictly for gamification, may benefit from greater provisions of positive feedback. In this study, it is possible that positive feedback reinforced the natural movement of the subject for this simple reach-to-touch task. Alternatively, negative feedback may have facilitated uncertainty in competence [70] despite task simplicity. This uncertainty may have drawn attention from the task such that perception of control, i.e., sense of agency, is expectantly impaired. Furthermore, the lack of significant change in agency or performance from zero suggests negative feedback negated any learning or confidence that would otherwise arise from repetitive task practice [59].

### 4.2 Agency was progressively increased during positive feedback training

Amplifying perception of success can precipitate greater confidence in motor learning and performance [71]. In this study, positive feedback produced increase in agency both intra-training and post-training from baseline. The progressive increase in agency during training was positive and significant (non-zero). Previous literature demonstrated how social comparison of performance can facilitate motor learning, even when provided bogus social-comparative feedback [72, 73]. Similarly, our reach-to-touch task was designed to be simple so that feedback was not relied upon for learning based upon true information about performance. Simple feedback was essentially provided as social-comparative reward or punishment to positively or negatively prime the subject. The effects of simple positive feedback appear to include facilitation of agency during training in addition to significant post-training retention. Negative feedback also produced a net increase in agency during training, but it was not significant from zero. As such, the effects of negative feedback on intra-training changes in agency was inconclusive.

### 4.3 Reaching performance progressively decreased during positive feedback training

The actual reduction in performance for minimizing path length during positive feedback training may be attributed to loss of focus and attention. Reward has been shown to modulate attention independent of task action [74]. In our study, the preponderance of positive affirmation without clear self-indication of improved behavior may have shifted attention away from motor performance and towards appetite for the dopaminergic reward response during training. However, positive feedback still produced the desired post-training effect of improved performance compared to baseline. While apparently contradictory, this post-training improvement further suggests that positive feedback did not facilitate intrinsic motor learning, but rather primed post-training performance as intended. This paradox with progressive decrease in training performance but improved post-training outcomes was apparently specific to positive feedback. Performance results for negative feedback were effectively neutral (not significantly different from zero) for both training slope and post-training changes. This suggests that the positive feedback had unique effects during and after training that is independent of how the feedback was presented. As such, we might discount effects of superstition [75] due to provision of feedback not necessarily indicative of actual performance.

We postulate that post-training effects with positive feedback in this study was not a matter of learning but increased confidence because the motor task was simple. The task itself was simple enough to learn and perform successfully with minimal practice, which allowed training to mainly prime perception of movement. Priming can positively affect agency [67], and it appears increased agency can then promote better performance of a simple reach-touch task in VR. Isolating the effect of perception from learning with reward may be explained by the increased amplitude of the P300 component in the event-related brain potential. The P300 component endogenously signifies personal reaction to an event and is correlated with reward, regardless of value [76]. In our study, feedback value would be signified by whether it truly indicated performance better or worse than average. Furthermore, previous studies also suggest reward positivity amplitude to be unrelated to trial-to-trial behavioral adjustments in task performance [77].

### 4.4 Positive feedback reduced time to contact but had no clear effect on contact accuracy

Both ‘contact’ performance metrics were known to be secondary to minimizing reach path length in this study. However, these secondary goals were necessary to: 1) constrain and contextualize the performance task, i.e., minimize reach while accurately aiming for a contact target; and 2) enforce control responsibility, i.e., pace the movement prior to contact to better assess perception of movement control. While positive feedback clearly demonstrated increases in agency and reaching performance, the effects on the secondary performance metrics of contact were mixed.

Even prior to training, subjects demonstrated high capability to achieve the target contact time of 3000 msec. Thus, the bandwidth to improve contact timing was narrow whereby training had little effect in improvement. However, there was a significant general reduction in contact timing from baseline with positive feedback. A plausible explanation for the PF group completing reaches faster may be enhanced sense of self-efficacy with increased perception of success and capability [70]. Reduced contact time may also result with heightened expectancy of reward and faster movements due to positively validated past experience [78]. Enhanced agency may also play a role through priming a faster motor responses [79].

### 4.5 Agency had positive correlation to internality with positive feedback training

There was significant positive covariance between I-score (internality) of the MHLC survey and post-training agency with positive feedback. This result countered our initial hypothesis that more extrinsically motivated individuals would be more affected by positive or negative feedback. Thus, internalized performance expectancies (based on social comparisons) may be tied to self-efficacy and a more intrinsically oriented personality [80, 81]. Higher internals may be inclined to stronger sense of agency with positive affirmation of performance. Positive performance expectancy enhances intrinsically driven behavior [18, 44, 46]. Therefore, highly internal participants may have been more receptive to positive affirmation in this study.

#### Limitations

The major limitations of this study include task simplicity, utilizing only binary feedback, and narrow recruitment of personality types. Functional tasks to be rehabilitated using virtual reality or conventional modes of therapy typically involve reach, grasp, and manipulation. Our intent in this study was to simplify the task (reach-to-touch) to minimize potential learning and demonstrate fundamental links across performance, agency, and positive feedback versus negative feedback. Future studies may incorporate more complex tasks but with similar metrics of performance such as efficiency, accuracy, and time-to-completion.

There may be a gradient in positivity of feedback that could be further identified to truly optimize rehabilitation that uses performance feedback for learning. Our provision of feedback was binary with only a single proportion for either largely positive (80% ‘GOOD’ messaging) or negative (80% ‘BAD’ messaging) feedback. For this study, we chose only to directly compare a single PF and single NF group. While there are intended implications of motor learning in our study, we reduced the scope of this investigation to effects of simple positive and negative tone on agency and performance. Additional testing of a ‘no feedback’ group would discriminate the added learning benefit of positive feedback versus practice only. However, we believe the simplicity of our experimental task appropriately narrowed the scope of this study to strictly observe the effects of simple positive and negative tone on performance and agency.

For a more complex task, learning can be more prevalent and subtle changes in performance could be independently discerned by the subject. Thus, our predetermined provision of feedback would have been confusing and detrimental to learned performance for both PF and NF groups. Furthermore, while our provision of simple positive feedback appeared to improve performance after training, our presentation of negative feedback did not generate notable post-training changes. Thus, it is not clear whether our simple negative feedback was necessarily aversive, let alone a true mode of punishment adversely affecting movement performance. Given the apparent neutral impact on performance with simple negative feedback, the NF group may have effectively served as a ‘no feedback’ group. Studies specifically investigating the learning of complex tasks should consider appropriate controls to serve as a baseline from which learning factors may be better assessed [50]. Appropriate projection of complex function in VR may require additional marker clusters to animate individual digits and arm postures. To better promote learning of complex tasks, feedback could also be provided more quantitatively although qualitative expressions of performance, like social comparative constructs, are very motivating. Thus, investigating additional tiers of qualitative feedback (e.g., ‘excellent’, ‘very good’, ‘fair’) may be fruitful. Such studies could provide insight as to how the internal model updates to make predictions for execution due to probabilistic levels of reward of positivity in feedback.

Finally, we did not have the resources available to field participants that necessarily provided a broad range of MHLC personality scores. This inherently limited the conclusions we could make about how personality is a co-factor in accelerating reward-based movement agency and performance. With our limited set of survey responses, we still observed a significant positive relationship between internality and post-reward agency. This could motivate further experiments that can better consider how personality relates to agency and performance in VR rehabilitation.

#### Concluding remarks including recommendations

Our study incorporated simple binary feedback (GOOD/BAD messaging) to demonstrate greater agency and performance with positive feedback conditioning compared to negative feedback for a VR reach-to-touch task. It is likely that typical activation of dopamine pathways contributed to both agency and performance. It is still unclear how positive versus negative feedback may affect perception of movement history [82] and agency-based learning of complex tasks. Although reward has been shown to enhance motor memory compared to punished and neutral groups [83], to our knowledge, no other study has related positive feedback to sense of agency and VR movement. We suggest that experimental design features that strategically employs feedback tone for both improved agency and performance has greater potential to leverage cognitive engagement with VR rehabilitation. Additional work to pursue for potential clinical translation include: (1) testing complex tasks that are more functionally relevant, (2) characterizing agency and performance effects across a broad spectrum of positive and negative feedback, and (3) further considering the role of personality types.

It is still unclear how performance and agency may be co-modulated by feedback for more complex tasks in neuromotor rehabilitation. In this case, feedback would need to inform and motivate. However, these objectives may be separable as reward contingent on performance of a self-control task can deplete subsequent performance compared to non-contingent reward [84]. In our study, we largely employed non-contingent positive and negative feedback, but the contingency was unknown to the subjects. While true performance feedback is classically used to facilitate complex motor learning [85], our study suggests a preponderance of positive feedback can fundamentally increase performance and agency. We postulate that rehabilitation approaches should consider how to balance provision of informative feedback for learning with greater positivity, even with sub-par performance, to accelerate motor gains.

The implications for this study are mainly applicable to VR rehabilitation because of the versatility and customizability in providing feedback with computerized environments. Delivery of positive feedback to enhance agency and performance could still be employed for more conventional modes of physical rehabilitation. It is beyond the scope of this study to consider the effects of positive feedback on agency using a virtual hand avatar versus only a real hand. Thus, identifying how feedback positivity best enhances agency and performance may depend on the specific rehabilitation environment. However, the growing prevalence of VR in rehabilitation makes it a suitable environment to consider for this study.

Rehabilitation that customizes performance feedback is consistent with approaches advocating for greater personalization in clinical treatments [86]. While considering how best to present multiple levels of qualitative and quantitative feedback, a guiding design principle may be the potential response based on personality. Rehabilitation may be accelerated by thoughtfully delivering a mosaic of performance feedback based on specific characteristics of each person. These characteristics may include not only personality, but also nature of neural injury and current movement tendencies. Additionally, rehabilitation paradigms may be constructed to empower persons, say through agency, to progressively change performance tendencies over time. Studies that carefully dissect the interplay between physical and cognitive characteristics could open exciting new pathways to more efficient and effective neurorehabilitation.
